# 

**Published:** 1916-05-06

**Authors:** 


### Personalia.

Dr. J. M. H. MacLeod, M.D., C.M. (Aberd.), M.R.C.S. (Lond.), physician to the skin department at the Charing Cross Hospital, has been appointed by the Metropolitan Asylums Board as the consulting physician for skin diseases to their children's institutions, in place of Dr. James Galloway, who has been invited by the Government to undertake the duties of consulting physician to the British Expeditionary Force in France, and has been granted leave of absence by the Board.

### Matrons' and Sisters' Appointments.

Royal South Hants and Southampton Hospital.—Miss Nellie Duncombe, who was trained at this hospital and holds the certificate of the Incorporated Society of Trained Masseuses, has been appointed theatre sister.

St. Nicholas Hospital for Children, Birchington.—Miss Grace Brady, who has been assistant matron at the General Hospital, Merthyr Tydfil, has been appointed sister. She was trained at Ancoats Hospital, Manchester, and she has since done private, district, and surgical nursing at the Nurses' Institution and Nursing Home, Stoke-on-Trent.

York County Hospital.—Miss Maude Matthews has been appointed sister. Trained at Liverpool Royal Infirmary, she has since held various appointments—viz., at the Mount Vernon Hospital for Diseases of the Chest, at Bolton Infirmary, and at York County Hospital.

**NOTE.—Particulars of Appointments for insertion in this column will be welcomed.**

RATES OF SUBSCRIPTION: Twelve months, 6/6; Six months, 4/-; Three months, 2/-, post free to any address in the United Kingdom. Abroad, Twelve months, 8/8; Six months, 5/-; Three months, 2/6, post free.—Address The Subscription Dept., "THE HOSPITAL," The Hospital Building, 28/29 Southampton Street, Strand, London, W.C.

**Publications.**

**WORKS BY**

### Sir LAUDER BRUNTON, Bt., M.D., F.R.C.P., F.R.S., &c.

*Consulting Physician to St. Bartholomew's Hospital.*

### COLLECTED PAPERS ON CIRCULATION & RESPIRATION

**First Series. Experimental. 8vo. pp. 696, 7s. 6d.**
**Second Series. Chiefly Clinical. In the Press. 5s. net.**

MACMILLAN & CO., LTD., LONDON.

### LECTURES ON THE THERAPEUTICS OF THE CIRCULATION

**SECOND EDITION.**

**Small fcap. 8vo. Price 5s. net.** So much matter has been added to this edition that it is practically a new book. **Third Reprint.**

JOHN MURRAY, ALBEMARLE STREET, LONDON.

**Recently Published. Demy 8vo. 656 pp. Price 10s. 6d. net.**

### FOOD AND FEEDING IN HEALTH AND DISEASE.

**A MANUAL OF PRACTICAL DIETETICS.**

By CHALMERS WATSON, M.D., F.R.C.P.E., Assistant Physician, Royal Infirmary, Edinburgh; Editor of the "Encyclopædia Medica."
Second Edition, Revised.

ATHENÆUM.—"Dr. Chalmers Watson has done well to write a standard book on 'Food and Feeding,' for it is a long time since any satisfactory book on dietetics has appeared in the English language."

**Crown 8vo. 352 pages. Price 5s. net.**

### PRACTICAL GYNÆCOLOGY. A MANUAL FOR NURSES AND STUDENTS.

Being a Second and Enlarged Edition of GYNÆCOLOGICAL NURSING. By NETTA STEWART, Sister in the Extra-mural Gynæcological Wards of the Royal Infirmary, Edinburgh; and JAMES YOUNG, M.B., F.R.C.S.E., Clinical Tutor in Surgery and late Resident Gynæcologist, Royal Infirmary; Physician to Lauriston Pre-Maternity Home, Edinburgh. Numerous Illustrations and Plates.

"This is a book we can cordially and unreservedly recommend. The style of the work is excellent, and the subjects are handled with admirable discrimination."—EDINBURGH MEDICAL JOURNAL, April 1909.

OLIVER & BOYD. EDINBURGH: Tweeddale Court. LONDON: 33 Paternoster Row

By SIR WILLIAM BENNETT, K.C.V.O., F.R.C.S.

***Crown 8vo. 5s. net.***

### INJURIES AND DISEASES OF THE KNEE JOINT

With 34 Illustrations.

NISBET & CO.

***Also, 8vo. 6s.***

FOURTH EDITION.

**Massage and Movements in Recent Fractures; Sprains and their Consequences; Rigidity of the Spine and the Management of Stiff Joints.**

With 23 Illustrations.

